# Contiguous 2,2,4-triamino-5(2*H*)-oxazolone obstructs DNA synthesis by DNA polymerases *α*, *β*, *η*, **ι**, *κ*, REV1 and Klenow Fragment exo^−^, but not by DNA polymerase ζ

**DOI:** 10.1093/jb/mvv103

**Published:** 2015-10-21

**Authors:** Masayo Suzuki, Katsuhito Kino, Taishu Kawada, Takanori Oyoshi, Masayuki Morikawa, Takanobu Kobayashi, Hiroshi Miyazawa

**Affiliations:** ^1^Kagawa School of Pharmaceutical Sciences, Tokushima Bunri University, 1314-1, Shido, Sanuki, Kagawa 769-2193, Japan and; ^2^Faculty of Science, Department of Chemistry, Shizuoka University, 836 Ohya, Suruga, Shizuoka 422-8529, Japan

**Keywords:** 2,2,4-triamino-5(2*H*)-oxazolone, contiguous damage, DNA polymerase, oxidation of guanine, translesion synthesis

## Abstract

Guanine is the most easily oxidized of the four DNA bases, and contiguous guanines (GG) in a sequence are more readily oxidized than a single guanine in a sequence. Continued oxidation of GGs results in a contiguous oxidized guanine lesion. Two contiguous 2,5-diamino-4*H*-imidazol-4-ones, an oxidized form of guanine that hydrolyses to 2,2,4-triamino-5(2*H*)-oxazolone (Oz), are detected following the oxidation of GG. In this study, we analysed translesion synthesis (TLS) across two contiguous Oz molecules (OzOz) using Klenow Fragment exo^−^ (KF exo^−^) and DNA polymerases (Pols) *α*, *β*, *ζ*, *η*, *ι*, *κ* and REV1. We found that KF exo^−^ and Pols *α*, *β*, ι and REV1 inserted one nucleotide opposite the 3′ Oz of OzOz and stalled at the subsequent extension, and that Pol *κ* incorporated no nucleotide. Pol *η* only inefficiently elongated the primer up to full-length across OzOz; the synthesis of most DNA strands stalled at the 3′ or 5′ Oz of OzOz. Surprisingly, however, Pol *ζ* efficiently extended the primer up to full-length across OzOz, unlike the other DNA polymerases, but catalysed error-prone nucleotide incorporation. We therefore believe that Pol *ζ* is required for efficient TLS of OzOz. These results show that OzOz obstructs DNA synthesis by DNA polymerases except Pol *ζ*.

Mutations of genetic information are caused by various endogenous and exogenous oxidative stresses, leading to carcinogenesis, aging and other diseases. Guanine is the most sensitive of the four DNA bases to oxidative stress (1), and oxidized guanine lesions induce G:C-T:A and G:C-C:G transversions (2). These transversions are observed in many key genes in tumours, such as the *p53* tumour suppressor gene and the *K-ras* oncogene (3–5).

8-Oxo-7,8-dihydroguanine (8-oxoG) is a major oxidized guanine lesion produced under various oxidative conditions. 8-OxoG can form a Hoogsteen base pair with adenine (6), and this lesion causes G:C-T:A transversions in *Escherichia coli* (*E.coli*) and mammalian cells (7–9). Because of its lower oxidation potential, 8-oxoG is more susceptible to oxidation than guanine. The oxidation of guanine and the further oxidation of 8-oxoG produces 2,5-diamino-4*H*-imidazol-4-one (Iz) (10–13). We recently reported the detection of Iz in photooxidized single- and double-stranded DNA (14*, *15). Although Iz predominantly induces G:C-C:G transversions *in vitro* and *in vivo* (16*, *17), this lesion is slowly hydrolysed to 2,2,4-triamino-5(2*H*)-oxazolone (Oz) in neutral aqueous solution (Fig. 1A) (10). In addition, Oz has been detected in samples of liver DNA, and photooxidation in the presence of 5-methylcytosine significantly increased the yield of Oz at the CpG site in the *p53* tumour suppressor gene (18). Therefore, it appears that Oz has a more significant biological effect than Iz.

**Fig. 1:**
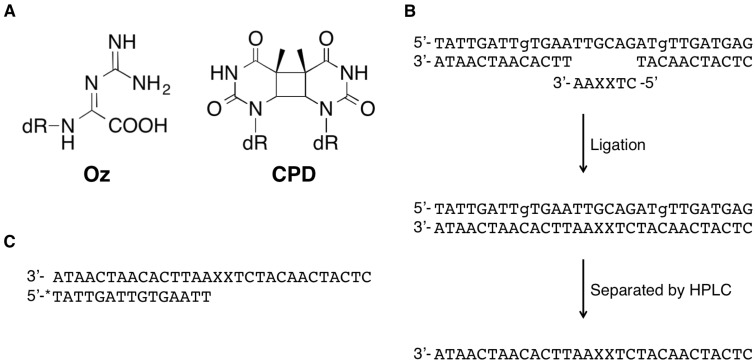
**Oligonucleotides used in the study**. (**A**) Structures of Oz and CPD. (**B**) Outline of the preparation of 30-mer DNA containing the OzOz lesion. XX represents OzOz. (**C**) The template and primer set used for analyses of extension past the OzOz lesion and insertion opposite this lesion (Figs 2–4, 5A and 5B). XX represents GG, OzOz or CPD. The 15-mer primer is labelled with Alexa 680, indicated by ‘*’.

We previously investigated which nucleotides are incorporated opposite a single Oz using Klenow Fragment exo^−^ (KF exo^−^), *Sulfolobus solfataricus* DNA polymerase (Pol) IV and the eukaryotic Pols *α*, *β*, *γ*, *δ*, *ε*, *ζ*, *η*, *ι*, *κ* and REV1 (19–21). We found that guanine was incorporated opposite the single Oz by all DNA polymerases (19–21) except REV1 which incorporated cytosine opposite the single Oz through its deoxycytidyl transferase activity (20). In addition, *ab initio* calculations led us to previously propose that Oz forms a stable, planar base pair with guanine via two hydrogen bonds (19*, *22). Recently, we reported that the Oz:G base pair is more thermodynamically stable than Oz:A, Oz:C and Oz:T (23), suggesting that Oz is most likely to cause G:C-C:G transversions. Moreover, we previously reported that human NEIL1 and NTH1 excise Oz from not only Oz:C but also Oz:G base pairs (24). Hence, when DNA polymerases incorporate guanine opposite the single Oz, G:C-C:G transversions caused by Oz would not be prevented even if these repair enzymes act on Oz.

Guanines in contiguous guanine sequences are more readily oxidized by a one electron oxidant compared with a single guanine due to their lower redox potential (25*, *26). Guanine-contiguous sequences exist in many important genomic regions, such as the *K-ras* oncogene and telomeres. The continued oxidation of contiguous guanines under high oxidation conditions results in a contiguous oxidized guanine lesion. In addition, guanines in a contiguous guanine sequence are oxidized by singlet oxygen (27). It is therefore necessary to analyse the influence of contiguous guanine damage on DNA replication. There have been several reports to date of the influence of two contiguous 8-oxoGs on DNA synthesis (28*, *29). In addition, we previously reported that two contiguous Izs are produced by the oxidation of two contiguous guanines in single- and double-stranded DNA (12), suggesting that hydrolysis of these Iz molecules will produce two contiguous Oz molecules (OzOz). Since Iz is an oxidation product of both guanine and 8-oxoG, the biological influence of OzOz is important and should be analysed.

Pols *α*, *γ*, *δ* and *ε* catalyse elongation to the full-length across a single Oz with high fidelity (19*, *20), indicating that a single Oz does not obstruct DNA synthesis. However, contiguous Oz molecules could stall DNA synthesis more effectively than a single Oz and thus represent more serious DNA damage than a single Oz. We here investigated the influence of contiguous Oz molecules on DNA synthesis by examining whether KF exo^−^, Pols *α*, *β*, *ζ*, *η*, *ι*, *κ* and REV1 can elongate the primer across OzOz. In addition, we analysed which nucleotide is incorporated opposite OzOz.

## Materials and Methods

### DNA substrates

A 30-mer DNA template containing OzOz (30-mer-OzOz) (5′-CTCATCAACATCTXXAATTCACAATCAATA-3′, where XX represents OzOz) was constructed in several steps (Fig. 1B). First, a 6-mer oligonucleotide containing GG (5′-CTGGAA-3′) was oxidized to a 6-mer oligonucleotide containing IzIz by UV irradiation (365 nm) in the presence of riboflavin using a previously described method (12), then the Iz molecules were hydrolysed to provide a 6-mer oligonucleotide containing OzOz (6-mer-OzOz) (5′-CTXXAA-3′, where XX represents OzOz) (10). Next, this 6-mer oligonucleotide and a 13-mer oligonucleotide (5′-TTCACAATCAATA-3′) were phosphorylated by T4 polynucleotide kinase (New England Biolabs, Ipswich, MA). The 30-mer-OzOz was prepared by ligation of an 11-mer oligonucleotide (5′-CTCATCAACAT-3′) to the 5′ side of the 6-mer OzOz and the phosphorylated 13-mer oligonucleotide to the 3′ side of the 6-mer-OzOz using T4 DNA ligase (Takara, Otsu, Japan), using a 30-mer DNA-RNA chimeric oligonucleotide (5′-TATTGATTgTGAATTGCAGATgTTGATGAG-3′, where g represents guanosine, not deoxyguanosine) as a template. Finally, 30-mer-OzOz was isolated by HPLC and confirmed by a time-of-flight mass spectrometer (Bruker Daltonics K.K., Yokohama, Japan).

DNA templates containing GG or cyclobutane pyrimidine dimer (CPD) (where XX represents GG or CPD) and Alexa 680-labelled 15-mer primer (5′-Alexa680-TATTGATTGTGAATT-3′) were purchased from Japan Bio Services Co., Ltd. (Saitama, Japan).

### Enzymes

Human Pols *η*, *ι* and *κ* were separately expressed in *E.coli* systems and purified as described previously (20*, *23). KF exo^−^ (Fermentas, Waltham, MA), calf thymus Pol *α* (CHIMERx, Milwaukee, WI), human Pol *β* (CHIMERx), yeast Pol *ζ* (Enzymax, Lexington, KY) and yeast REV1 (Enzymax) were purchased.

### DNA polymerase assays

Polymerization reactions (5 µl) were carried out using the following mixtures: (for KF exo^−^) 50 mM Tris-HCl (pH 8.0), 5 mM MgCl_2_, 1 mM DTT, 100 µg/ml BSA; (for Pol *α*) 40 mM Tris-HCl (pH 8.0), 5 mM MgCl_2_, 10 mM NaCl, 45 mM KCl, 10 mM DTT, 250 µg/ml BSA, 5% glycerol; (for Pol *β*) 50 mM Tris-HCl (pH 8.8), 10 mM MgCl_2_, 1 mM DTT, 400 µg/ml BSA; (for Pols *η*, *κ*, *ζ* and REV1) 50 mM Tris-HCl (pH 8.0), 2 mM MgCl_2_, 5 mM DTT, 100 µg/ml BSA; (for Pol *ι*) 40 mM Tris-HCl (pH 7.5), 8 mM MgCl_2_, 150 mM NaCl, 1 mM DTT, 10 µg/ml BSA, 10% glycerol. All reaction mixtures contained 100 fmol of the template and 50 fmol of 5′-Alexa680-labelled primer. The template and 15-mer primer are shown in Fig. 1C. Other conditions, and the concentrations of deoxyribonucleoside triphosphates (dNTPs) and DNA polymerases, are specified in the figure legends. Reactions were performed at 37°C for 30 min for KF exo^−^, Pols *α*, *β*, *η* and *κ*, at 30°C for 30 min for Pol *ζ* and REV1 and at 30°C for 15 min for Pol *ι*. All reactions were stopped by adding 5 µl of stop buffer (15 mM EDTA and 10% glycerol). Aliquots (2.5 µl) were subjected to electrophoresis in a denaturing 16% polyacrylamide gel containing 8 M urea at 30 W for 90 min. Gel images were quantified using an Odyssey Infrared Imaging System (LI-COR, Lincoln, NE).

## Results and Discussion

### Preparation of 30-mer DNA template containing the OzOz lesion

As previously described (12), 6-mer-OzOz was prepared via hydrolysis of 6-mer-IzIz oxidized from the corresponding 6-mer oligonucleotide containing GG, and the 30-mer-OzOz was prepared by the ligation of two oligonucleotides (11-mer and 13-mer) to the 5′ and 3′ side of 6-mer-OzOz, respectively (Fig. 1B).

We attempted to prepare 30-mer oligodeoxynucleotide as a template by ligating 11-mer and 13-mer oligonucleotides to the 5′ and 3′ side of 6-mer-OzOz but could not separate the 30-mer-OzOz and 30-mer template oligodeoxynucleotide by HPLC (data not shown). In contrast, the ligation of 11-mer and 13-mer oligonucleotides to 6-mer-OzOz provided 30-mer-OzOz and 30-mer DNA-RNA chimeric oligonucleotide that could be separated by HPLC. Thus, we used this 30-mer DNA-RNA chimeric oligonucleotide as a template for ligation.

### Stalling of DNA synthesis by KF exo^−^, calf thymus Pol *α* and human Pol *β* at the OzOz lesion

**Fig. 2:**
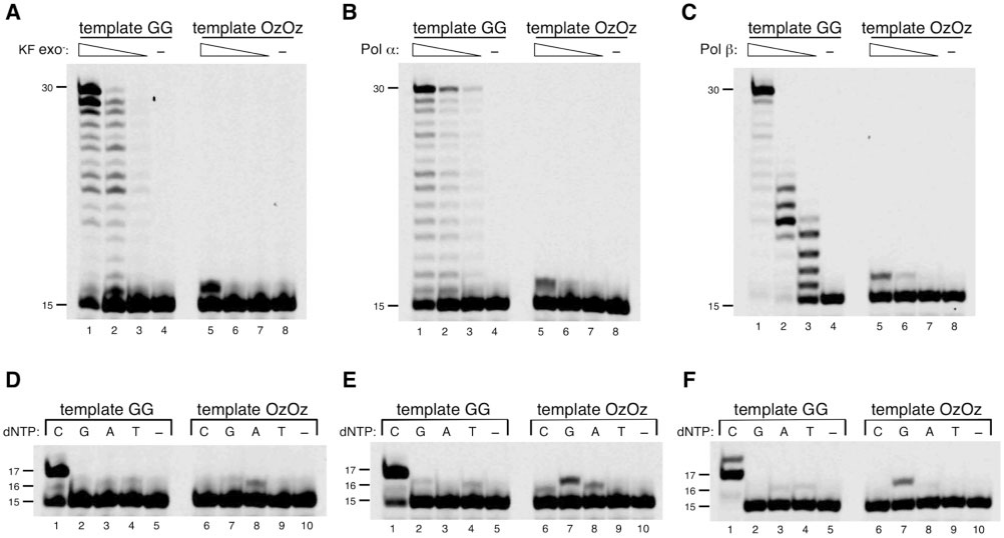
**DNA synthesis by KF exo**^−^, **calf thymus Pol α or human Pol β across OzOz.** (**A–C**) Primer extension across OzOz by KF exo^−^ (A), Pol α (B) or Pol β (C). Decreasing amounts of KF exo^−^ (250 µU in lanes 1 and 5, 25 µU in lanes 2 and 6, 2.5 µU in lanes 3 and 7), Pol α (100 mU in lanes 1 and 5, 33 mU in lanes 2 and 6, 10 mU in lanes 3 and 7) or Pol β (25 mU in lanes 1 and 5, 2.5 mU in lanes 2 and 6, 250 µU in lanes 3 and 7) was incubated with template (containing GG in lanes 1–4, containing OzOz in lanes 5–8) and 100 µM of each of the four dNTPs. Lanes 4 and 8 contained no enzyme as negative controls. (**D–F**) Nucleotide selectivity of KF exo^−^ (D), Pol α (E) or Pol β (F) opposite OzOz. KF exo^−^ (250 µU), Pol α (100 mU) or Pol β (2.5 mU) was incubated with template (containing GG in lanes 1–5, or OzOz in lanes 6–10) and 100 µM of a single dNTP (N = C, G, A or T) (lanes 1–4 and 6–9). Lanes 5 and 10 contained no enzyme as negative controls.

We examined whether KF exo^−^, and Pols *α* and *β*, can elongate the primer across the OzOz lesion by synthesizing a 30-mer DNA oligonucleotide containing the OzOz lesion and using it as template. DNA template containing two undamaged guanines (GG) was readily elongated by KF exo^−^, as well as by Pols *α* and *β*, to produce full-length product (Fig. 2A–C, lanes 1–3). In contrast, KF exo^−^, and Pols *α* and *β* incorporated only one nucleotide opposite the 3′ Oz of the OzOz lesion and could not continue DNA synthesis after this insertion (Fig. 2A–C, lane 5).

The nucleotide incorporated opposite the 3′ Oz of the OzOz lesion was identified using DNA polymerase assays in the presence of only one deoxyribonucleoside triphosphate (dCTP, dGTP, dATP or dTTP). KF exo^−^ preferentially incorporated dATP opposite the 3′ Oz of the OzOz lesion (Fig. 2D, lane 8), Pol *α* incorporated dGTP opposite the 3′ Oz of the OzOz lesion more efficiently than dATP or dCTP (Fig. 2E, compare lane 7 with lanes 6 and 8), and Pol *β* predominantly incorporated dGTP (Fig. 2F, lane 7). We previously demonstrated that KF exo^−^ incorporates dGTP and dATP opposite a single Oz, and Pols *α* and *β* insert dGTP opposite a single Oz. Thus, the tendency for KF exo^−^ and Pols *α* and *β* to incorporate dGTP or dATP opposite the 3′ Oz of the OzOz lesion is similar to their tendency when incorporating a nucleotide opposite a single Oz (19).

KF exo^−^, Pols *α* and Pols *β* are DNA repair or DNA replication polymerases (30) and therefore exhibit moderate to high fidelity. In general, DNA synthesis by KF exo^−^, Pols *α* and Pols *β* is easily obstructed by various lesions. Although a single Oz did not stall their DNA synthesis, as we previously reported (19), two contiguous Oz molecules did stall their DNA synthesis, indicating that the latter impart more serious damage to DNA polymerases during DNA replication and repair.

### Stalling of DNA synthesis by human Pol *ι*, human Pol *κ* and yeast REV1 at the OzOz lesion

**Fig. 3:**
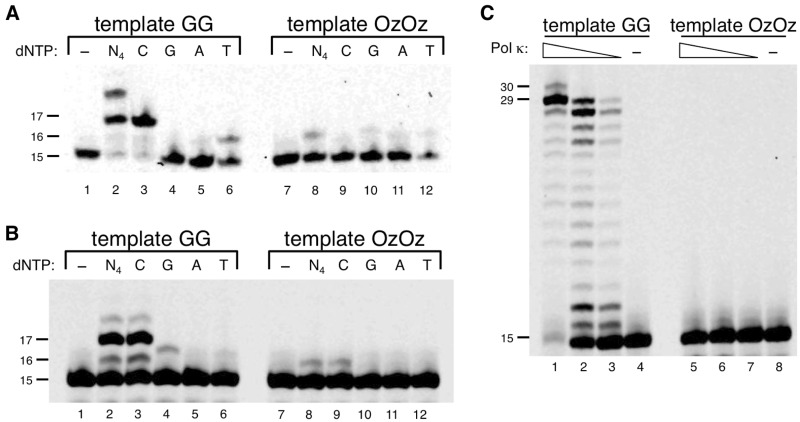
**DNA synthesis with human Pol ι, human Pol κ or yeast REV1 across OzOz.** (**A, B**) Primer extension across OzOz and nucleotide incorporation opposite OzOz by Pol ι (A) or REV1 (B). Pol ι (0.5 µg) or REV1 (4.2 ng) was incubated with template (containing GG in lanes 1–6, containing OzOz in lanes 7–12) and 100 µM of each of the four dNTPs (lanes 2 and 8) or 100 µM of a single dNTP (N = C, G, A or T) (lanes 3–6 and 9–12). Lanes 1 and 7 contained no enzyme as negative controls. (**C**) Primer extension across OzOz by Pol κ. Decreasing amounts of Pol κ (31 ng in lanes 1 and 5, 9.2 ng in lanes 2 and 6, 3.1 ng in lanes 3 and 7) were incubated with template (containing GG in lanes 1–4, containing OzOz in lanes 5–8) and 100 µM of each of the four dNTPs. Lanes 4 and 8 contained no enzyme as negative controls.

We also examined the ability of Pols *ι*, *κ* and REV1 to elongated past OzOz. Our recent study showed that Pol *ι* and REV1 incorporate only one nucleotide opposite a single Oz and are unable to catalyse translesion synthesis (TLS) past a single Oz (20). We therefore expected that a template containing the OzOz lesion would cause Pol *ι* and REV1 to incorporate only one nucleotide opposite the 3′ Oz of the OzOz lesion and stall at the subsequent nucleotide incorporation opposite the 5′ Oz of the OzOz lesion, regardless of their concentration. In fact, Pol *ι* and REV1 efficiently incorporated dCTP opposite both the 3′ G and 5′ G of the GG site (Fig. 3A and B, lane 3) but essentially could not incorporate even one nucleotide opposite the 3′ Oz of the OzOz lesion (Fig. 3A and B, lane 8). Pol *κ* incorporated essentially no nucleotide opposite OzOz and generated no detectable bypass products, even in the presence of excess enzyme (Fig. 3C, lanes 5–7).

We analysed which nucleotide is incorporated by Pol *ι* and REV1 opposite the 3′ Oz of the OzOz lesion and found that Pol *ι* slightly incorporated dGTP, dATP or dTTP (Fig. 3A, lanes 9–12) whereas REV1 incorporated dCTP with low efficiency (Fig. 3B, lanes 9–12). Although the efficiency of nucleotide incorporation opposite the 3′ Oz of the OzOz lesion by each polymerase was lower than for template containing GG, the tendency was consistent with incorporation opposite a single Oz.

Pols *ι*, *κ* and REV1 are classified as TLS polymerases (30*, *31), which play an important catalytic role in bypassing DNA lesions when DNA lesions stall replicative DNA polymerases. We recently showed that Pols *ι*, *κ* and REV1 can moderately incorporate nucleotide opposite a single Oz, and Pol *κ* can elongate the primer to nearly full-length beyond a single Oz (20). Nevertheless, DNA synthesis by Pols *ι*, *κ* and REV1 stalled at or before the 3′ Oz of the OzOz lesion, indicating that two contiguous Oz molecules likely cause serious damage by obstructing DNA synthesis catalysed by both replicative DNA and some TLS polymerases.

### TLS across the OzOz lesion and nucleotide incorporation by human Pol *η*

**Fig. 4:**
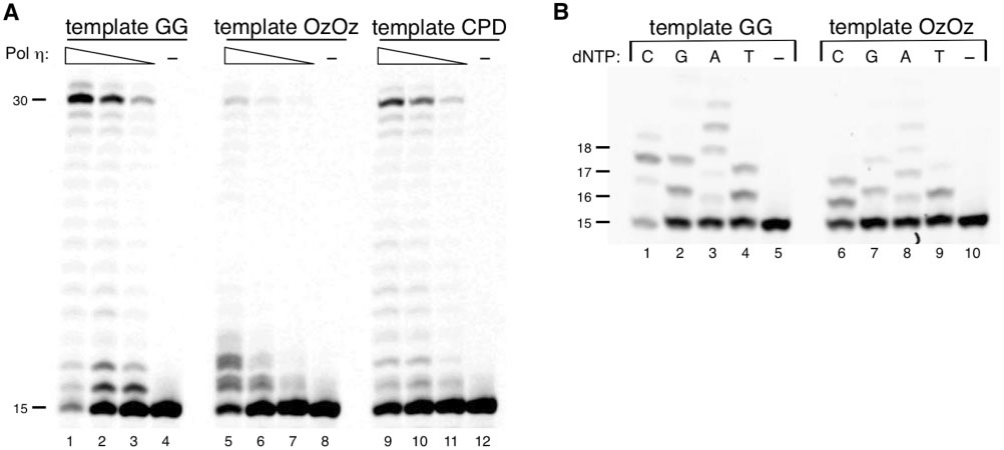
**DNA synthesis with human Pol η across OzOz**. (**A**) Primer extension across OzOz by Pol η. Decreasing amounts of Pol η (12 ng in lanes 1, 5 and 9, 4.0 ng in lanes 2, 6 and 10, 1.2 ng in lanes 3, 7 and 11) were incubated with template (containing GG in lanes 1–4, containing OzOz in lanes 5–8, containing CPD in lanes 9–12) and 100 µM of each of the four dNTPs. Lanes 4, 8 and 12 contained no enzyme as negative controls. (**B**) Nucleotide selectivity of Pol η opposite OzOz. Pol η (4.0 ng) was incubated with template (containing GG in lanes 1–5, and OzOz in lanes 6–10) and 100 µM of a single dNTP (N = C, G, A or T) (lanes 1–4 and 6–9). Lanes 5 and 10 contained no enzyme as the negative control.

Pol *η* is also a TLS polymerase (30) and can efficiently and accurately elongate past the CPD (32) that distorts template DNA, as well as moderately elongate the primer across a single Oz with reasonable efficiency (21). We therefore considered that Pol *η* may be able to catalyse past an OzOz lesion that stalls DNA synthesis by KF exo^−^, and Pols *α*, *β*, *ι*, *κ* and REV1. Analysis and comparison of the elongation efficiency by Pol *η* beyond GG, CPD and OzOz showed that Pol *η* efficiently elongated the primer up to full-length past GG and CPD (Fig. 4A, lanes 1–3 and 9–10) but inefficiently elongated the primer up to full-length past OzOz: most of the DNA synthesis by Pol *η* stalled at the 3′ Oz or 5′ Oz of the OzOz lesion (Fig. 4A lanes 5–7).

Pol *η* incorporated dCTP dGTP, dATP or dTTP opposite the 3′ G and 5′ G of GG (Fig. 4B, lanes 1–4). This trend remained even when the template contained the OzOz lesion (Fig. 4B, lanes 6–9), but the incorporation efficiency was lower, showing that nucleotide incorporation by Pol *η* is error-prone when replicating both OzOz and GG.

Our results showed that elongation past the OzOz lesion is very difficult even with Pol *η* which can bypass distorted templates such as CPD. However, Pol *η* can insert nucleotides opposite not only the 3′ Oz, but also the 5′ Oz of the OzOz lesion, unlike KF exo^−^, and Pols *α*, *β*, *ι*, *κ* and REV1. Therefore, even if DNA replication is stalled at the OzOz lesion, it is possible that DNA replication can restart following nucleotide insertion opposite the OzOz lesion by Pol *η*·

### TLS across the OzOz lesion and nucleotide incorporation by yeast Pol *ζ*

**Fig. 5:**
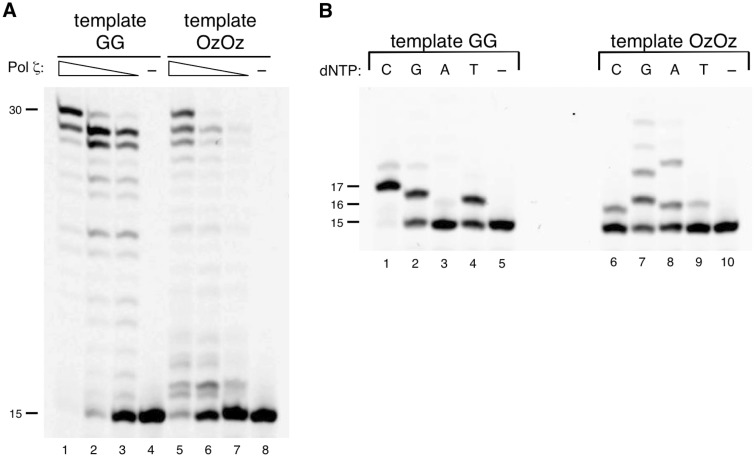
**DNA synthesis with yeast Pol ζ across OzOz.** (**A**) Primer extension across OzOz by Pol ζ. Decreasing amounts of Pol ζ (50 ng in lanes 1 and 5, 17 ng in lanes 2 and 6, 5.0 ng in lanes 3 and 7) were incubated with template (containing GG in lanes 1–4, containing OzOz in lanes 5–8) and 100 µM of each of the four dNTPs. Lanes 4 and 8 contained no enzyme as negative controls. (**B**) Nucleotide selectivity of Pol ζ opposite OzOz. Pol ζ (17 ng) was incubated with template (containing GG in lanes 1–5, and OzOz in lanes 6–10) and 100 µM of a single dNTP (N = C, G, A or T) (lanes 1–4 and 6–9). Lanes 5 and 10 contained no enzyme as negative controls.

We investigated whether Pol *ζ* stalls at the OzOz lesion similar to the other DNA polymerases, or if it can elongate past this lesion. Surprisingly, Pol *ζ* could efficiently elongate the primer up to full-length across OzOz, unlike the other DNA polymerases (Fig. 5A, lanes 5–7) but with lower efficiency compared with that across GG (Fig. 5A, compare lanes 6–7 with lanes 2–3).

Replication of template containing an undamaged GG by Pol *ζ* resulted in the generation of primer up to 17-mer in the presence of dCTP (Fig. 5B, lanes 1) but dGTP and dTTP were also inserted opposite the 3′ G of GG (Fig. 5B, lanes 2 and 4). In contrast, when the template contained the OzOz lesion, Pol *ζ* incorporated dGTP opposite both the 3′ Oz and 5′ Oz (Fig. 5B, lanes 7) and the incorporation of dCTP and dTTP was markedly decreased in comparison with the template containing GG (Fig. 5B, compare lanes 6 and 9 with lanes 1 and 4), whereas the incorporation of dATP was increased (Fig. 5B, compare lane 8 with lane 3). These results demonstrated that Pol *ζ* exhibited the same trend, catalysing error-prone nucleotide incorporation opposite the OzOz lesion and opposite a single Oz (20).

Pol *ζ* is a TLS polymerase whose activity depends on the type of DNA damage, and it plays an especially critical role in UV-induced mutagenesis (33–35). We recently reported that Pol *ζ* can elongate beyond a single Oz with approximately the same efficiency as that beyond a single G (20). Our current findings relating to elongation past the OzOz lesion strongly suggest that Pol *ζ* is an important enzyme for TLS in relation to Oz, regardless of whether the Oz is single or contiguous.

## Conclusion

KF exo^−^ and Pols *α*, *β*, *ι* and REV1 inserted only one nucleotide opposite the 3′ Oz of the OzOz lesion and with lower efficiency than that opposite undamaged guanine. They were strongly blocked at the subsequent insertion opposite the 5′ Oz of the OzOz lesion. The type of nucleotide incorporated opposite the 3′ Oz of the OzOz lesion was different for each DNA polymerase. No nucleotide incorporation opposite the 3′ Oz of the OzOz lesion by Pol *κ* was detected, indicating that DNA synthesis by Pol *κ* is completely inhibited by the OzOz lesion. These results showed that two contiguous Oz’s are likely to stall DNA synthesis by many DNA polymerases, including some TLS polymerases.

In contrast, Pol *η*, which catalyses efficient elongation across the CPD, elongated the primer up to full-length across the OzOz lesion with modest efficiency, but most DNA synthesis by Pol *η* stalled at the 3′ or 5′ Oz of the OzOz lesion. Therefore, two contiguous Oz’s cause serious damage by obstructing DNA synthesis, rather than by inducing mutagenesis, and two contiguous Oz molecules cause much more serious damage than a single Oz.

Surprisingly, unlike the other DNA polymerases, Pol *ζ* was able to extend the primer up to full-length across the OzOz lesion with error-prone nucleotide incorporation. We have therefore demonstrated for the first time the potential of Pol *ζ* as a DNA polymerase capable of efficiently catalysing the TLS of the OzOz lesion. Our results suggest that recruitment of Pol *ζ* to the OzOz lesion, even when other DNA polymerases are stalled at this lesion site, may prevent stalling of DNA replication at the OzOz lesion. Further work is needed to clarify the detailed mechanism of TLS relating to OzOz.

## Abbreviations

CPD: cyclobutane pyrimidine dimer
dNTP: deoxyribonucleoside triphosphate
Iz: 2,5-diamino-4*H*-imidazol-4-one
KF exo^−^: Klenow Fragment exo^−^
8-oxoG: 8-oxo-7,8-dihydroguanine
Oz: 2,2,4-triamino-5(2*H*)-oxazolone
Pol: DNA polymerase
TLS: translesion synthesis
